# The Risa R/Bioconductor package: integrative data analysis from experimental metadata and back again

**DOI:** 10.1186/1471-2105-15-S1-S11

**Published:** 2014-01-10

**Authors:** Alejandra González-Beltrán, Steffen Neumann, Eamonn Maguire, Susanna-Assunta Sansone, Philippe Rocca-Serra

**Affiliations:** 1 Oxford e-Research Centre, University of Oxford, Oxford, OX1 3QG, UK; 2 Leibniz Institute of Plant Biochemistry, Department of Stress and Developmental Biology, Halle, 06120, Germany

## Abstract

**Background:**

The ISA-Tab format and software suite have been developed to break the silo effect induced by technology-specific formats for a variety of data types and to better support experimental metadata tracking. Experimentalists seldom use a single technique to monitor biological signals. Providing a multi-purpose, pragmatic and accessible format that abstracts away common constructs for describing **I***nvestigations*, **S***tudies *and **A***ssays*, ISA is increasingly popular. To attract further interest towards the format and extend support to ensure reproducible research and reusable data, we present the **Risa **package, which delivers a central component to support the ISA format by enabling effortless integration with **R**, the popular, open source data crunching environment.

**Results:**

The **Risa **package bridges the gap between the metadata collection and curation in an ISA-compliant way and the data analysis using the widely used statistical computing environment **R**. The package offers functionality for: *i*) parsing ISA-Tab datasets into **R **objects, *ii*) augmenting annotation with extra metadata not explicitly stated in the ISA syntax; *iii*) interfacing with domain specific **R **packages *iv*) suggesting potentially useful **R **packages available in Bioconductor for subsequent processing of the experimental data described in the ISA format; and finally *v*) saving back to ISA-Tab files augmented with analysis specific metadata from **R**. We demonstrate these features by presenting use cases for mass spectrometry data and DNA microarray data.

**Conclusions:**

The **Risa **package is open source (with LGPL license) and freely available through Bioconductor. By making **Risa **available, we aim to facilitate the task of processing experimental data, encouraging a uniform representation of experimental information and results while delivering tools for ensuring traceability and provenance tracking.

**Software availability:**

The **Risa **package is available since Bioconductor 2.11 (version 1.0.0) and version 1.2.1 appeared in Bioconductor 2.12, both along with documentation and examples. The latest version of the code is at the development branch in Bioconductor and can also be accessed from GitHub https://github.com/ISA-tools/Risa, where the issue tracker allows users to report bugs or feature requests.

## Introduction

Recent breakthroughs in molecular biology techniques, from DNA microarray to mass-spectrometry and high-throughput sequencing have resulted in a huge surge in the availability of bioscience data. The data, however, cannot be understood, let alone re-used, without the availability of a description of the experimental context. This experimental context, usually written down in laboratory notebooks, often struggles to surface in digital form, failing to back up the associated raw data. Consequently, data management has become increasingly critical, involving tasks such as data curation, processing, analysis and storage in order to safeguard the datasets jointly with their contextual information. This contextual information, or *metadata*, encompasses description of samples, their source and features, the technologies used, the description of the experimental factors and response variable(s), the design of the experiment, the instrument parameters, the methods used for analysis and so on. This information is crucial to enabling the understanding, sharing, interoperability and re-use of the datasets for subsequent investigations. The inclusion of the metadata for data sharing and re-use is a requirement for an increasing number of research funders and journals.

However, the scientific communities behind each of the technologies (e.g. microarray, sequencing, flow-cytometry, mass spectrometry, Nuclear Magnetic Resonance or NMR spectroscopy) and the international repositories for *'omics *data have grown organically, developing and adopting technology-centric submission formats, data models and terminologies/ontologies for data annotation (see Table 1 for some examples of these elements per technology type; more details about each of these formats and terminologies can be found in the BioSharing catalogue [1]). Such a fragmented organisation fails to efficiently serve scientists, who, more often than not, use a range of techniques to acquire biological signals. This is evident at publication time, when datasets may have to be broken down and spread across technology-centric public repositories, causing *i*) duplication of effort when reporting shared elements and *ii*) loss of information by severing relations between datasets.

**Table 1:** Standards used in 'omics public repositories

Technology Type	Minimum Information Guidelines	Metadata Format	Ontology or Controlled Vocabulary	Public Repositories
DNA microarray*^a^*	MIAME [37]	MAGE-Tab [44]	MGED [45]	ArrayExpress [7], GEO [46] (via ArrayExpress*^b^*)

next generation sequencing	MIMARKS, MIxS [47]	SRA-XML	internal to SRA-schema	SRA, ENA

mass spectrometry	MIAPE [48]	PRIDE-XML	MS	PRIDE*^c ^*[9]

mass spectrometry, NMR spectroscopy	CIMR*^d^*	ISA-Tab	OBI [43], PSI-MS	Metabolights [10]

Some examples of formats, data models, terminologies and public repositories for omics experiments

*^a. ^*Both repositories, ArrayExpress and GEO, were originally designed to store DNA microarray data. While nowadays, they also allow submission of next generation sequencing data, these are done through underlying submissions to ENA (see http://www.ebi.ac.uk/training/online/course/arrayexpress-submitting-data-using-mage-tab/submission-hts-data) and SRA

*^b. ^*The GEO database does not deal with the MAGE-Tab format, but data from GEO can be accessed in ArrayExpress exposed in MAGE-Tab.

*^c. ^*PRIDE submission guidelines: http://www.ebi.ac.uk/pride/submissionGuidelines.do

*^d. ^*CIMR: http://msi-workgroups.sourceforge.net/

The ISA-Tab format was designed as a generic tabular format for the description of biological experiments [2], with focus on, but not limited to, experiments using high-throughput technologies. It relies on a hierarchical abstraction of the metadata about experiments. At the root of the metadata tree is the *Investigation *file, which aggregates a description of the overall experimental work, including the relevant publications and contacts. An *Investigation *can have one or more *Studies*. Each *Study *file contains metadata about the subjects under study, their attributes and any treatment applied to them. Each *Study *may define one or more *Assays *covering the response variables and defined as a pair of measurement type and the technology type. For instance, metabolite profiling (the measurement type) could be performed using mass spectrometry or NMR spectroscopy (the technology type). Each *Assay *file, in turn, compiles the information detailing how raw and derived data is produced from each study sample and points to associated data files.

A set of open source software tools, which are available from the GitHub platform for social coding [3], collectively referred to as the ISA infrastructure [2], support the manipulation of the format. Each of these tools is targeted to different users. The *ISAcreator Configurator *is aimed at curators and data managers, and allows for customisation of the ISA-Tab format with specific fields and ontology annotations through creation of configuration files in eXtensible Markup Language (XML) format. These XML files are designed to fulfil the requirements of one or more minimum information checklists [4], when relevant. *ISAcreator *and *OntoMaton *[5] are configuration-aware experimental metadata editors, supporting ontology-based annotations relying on NCBO Bioportal web services [6]. The former is a Java-based desktop application while the latter is based on Google-spreadsheets and thus, supports collaborative editing. Other modules support validation, storage, querying and filtering on a web application and conversion to specific formats required by public repositories [2]. ISA supports the move from paper-based laboratory notebooks to a spreadsheet-like tabular format, which can be converted automatically to the formats required by existing public repositories such as ArrayExpress [7], the European Nucleotide Archive (ENA) [8] and the Proteomics Identification Database (PRIDE) [9]. ISA-Tab is accepted, as is, in repositories such as Metabolights [10].

The ISA infrastructure users, collaborators and developers work jointly towards interoperable bioscience data [11] and are grouped into the *ISA Commons *[12]. They range from international public repositories and institutional repositories to funded research consortia and data journals, such as GigaScience [13] (by BioMed Central and BGI Shenzhen) and Scientific Data [14] (by Nature Publishing Group).

To reward experimental metadata and data collection, it is essential to ease the access to well-established analysis platforms. The **R **[15] environment for statistical computing is one such resource and it is particularly popular for the analysis of high-throughput, data heavy experiments through the Bioconductor (or BioC) project [16]. Built upon **R**, Bioconductor offers software and data packages for the comprehension, annotation, visualisation and analysis of high-throughput biological data. Furthermore, owing to an open source and open development policy, Bioconductor enjoys a very active user community.

To facilitate the integration of metadata in the experimental workflow and to bridge the gap between the ISA-compliant data collection and curation and the data analysis phases, we created the **Risa R**/Bioconductor package [17]. The package not only provides generic methods to parse and save ISA-Tab metadata, it also offers functionality to build specific, metadata informed objects, readily available for subsequent analysis with **R **packages specific for each domain. Therefore, it creates added value to the data collection and curation task, supporting analysis in a transparent way. It also offers unique means to perform data integration while enabling reproducible handling of experimental data and processed results. We believe this functionality is central to supporting provenance tracking of the metadata in bioscience experiments.

## The Risa package

The typical information payload found in experimental metadata consists of descriptors for biological materials and their properties (phenotypic information, provenance, and so on), processes and perturbations applied to those materials and links to data files produced by instruments recording a particular aspect of biological signals. These signals need to be processed, possibly relying on experimental design information, with the hope of extracting insightful new facts about biology.

The Biobase package [16], which is central to the Bioconductor framework, defines a set of core, standardised data structures to encapsulate and manipulate the experimental information outlined above. The remainder of the BioC packages build on top of Biobase to develop specialised capabilities geared towards data processing, statistical analysis and visualisation.

Therefore, one of the goals of the **Risa **package is to build the essential **R **functions and data structures from the Biobase package, extracted from ISA-Tab coded functional genomics experimental information. These data structures can then be channeled to dedicated analysis pipelines, tailored to meet the processing requirements of given technologies (e.g. for microarray processing [18], sequencing [19], mass spectrometry and flow cytometry [20]).

Data analysis using **R**/Bioconductor packages on ISA-Tab compliant data can therefore follow, relying on domain specific BioC packages. The initial version of the package, **Risa **1.0.0 [21], appeared in Bioconductor version 2.11 (R-2.15). Several improvements and extensions have already been implemented in Bioconductor version 2.12 [22]. The latest version of the code can be accessed in Bioconductor development branch and from the ISA-tools GitHub repository [3], where the issue tracker allows users to report bugs and feature requests.

### Supporting data with ISA-Tab metadata

**Figure 1:**
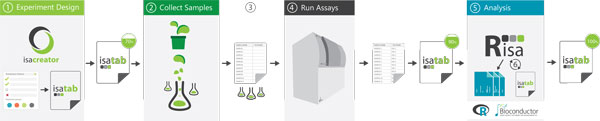
**ISA-guided domain specific workflows**. A possible workflow for an ISA-Tab augmented experiment design and execution. ① The experiment is designed with e.g. the ISAcreator to define the samples. ② The experiment is performed and samples are collected. ③ The sample names are transferred to the machine, e.g. a mass spectrometer (MS), to run the assays. ④ Assays are performed. In the MS example, it often occurs that the MS instrument software allows to copy & paste into its sample table, and creates a report (including MS filenames) that can go into the ISA-Tab assay information. ⑤ Domain specific **R **packages, such as *xcms *for MS, process the raw data. ⑥ The **Risa **objects are augmented with the results of the assay and the completed ISA-Tab dataset can be written back to disk.

In this section, we describe some ISA-Tab datasets, comprising different types of assays, that have been used to demonstrate the use of the **Risa **functionality. The typical workflow for use of **Risa **along the experimental steps is shown in Figure 1.

The faahKO[23] experimental data package [24] has been augmented to include an ISA-Tab metadata representation since its BioC version 1.2.11, and is used to demonstrate the **Risa **package features and constitutes the basis of examples for mass spectrometry data in the present manuscript. A compressed version of the metadata is also available within the **Risa **package itself.

The faahKO package comprises a subset of the data generated over the course of a global metabolite profiling study by Saghatelian *et al*. [25] in a classic two-condition, treated or untreated, study design. The data contains liquid chromatography/mass spectrometry (LC/MS) spectra from the spinal cords isolated from 6 wild-type and 6 fatty acid amide hydrolase (FAAH) knockout mice and corresponds to positive ionisation data. Those spectra are recorded in the Network Common Data Form (NetCDF) file format, amenable to manipulation as an *xcmsSet *(from the *xcms ***R**/Bioconductor package) holding detected peaks.

Additionally, the **Risa **package contains a compressed file with the metadata for the study ARMSTRONG-S-3 [26,27], as available in the Stem Cell Discovery Engine (SCDE) [28] repository. This dataset was produced when investigating the influence of the Wnt/beta-catenin pathway on the development of leukemia stem cells (LSCs) in acute myelogenous leukemia (AML). The study performed transcription profiling using DNA microarray in mouse models of LCSs induced AML either by co-expression of the Hoxa9 and Meis1a oncogenes or by the fusion oncoprotein MLL-AF9. The raw and processed data are available at the SCDE repository. This metadata is used to demonstrate the **Risa **functionality to deal with microarray data.

Datasets available from the Metabolights repository include ISA-Tab metadata. The **Risa **package was applied to one of these datasets [29] for data processing of mass spectrometry data and the script is available as a supplementary material in [10].

### Risa key features

#### Parsing ISA-Tab datasets

I/O operations represent the core of the functionality as expected for such a package. ISA-Tab datasets can be parsed with the readISAtab function given either a folder or a compressed archive. In addition, the package provides several persistence functions: persistence of a complete ISA-Tab dataset or updates to individual files under the same ISA-Tab dataset.

**Figure 2:**
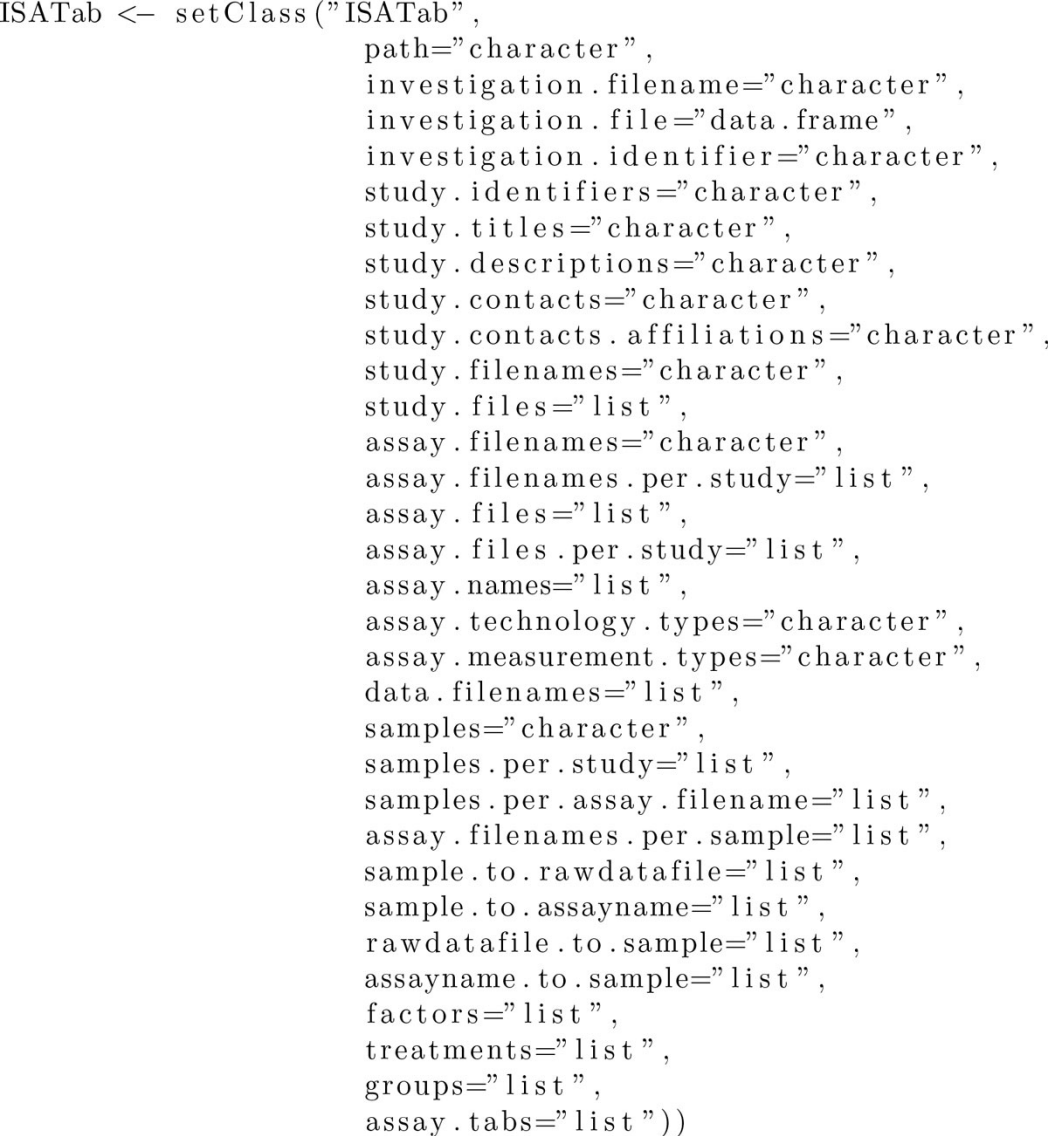
**Code for the ISATab-class**.

**Figure 3:**
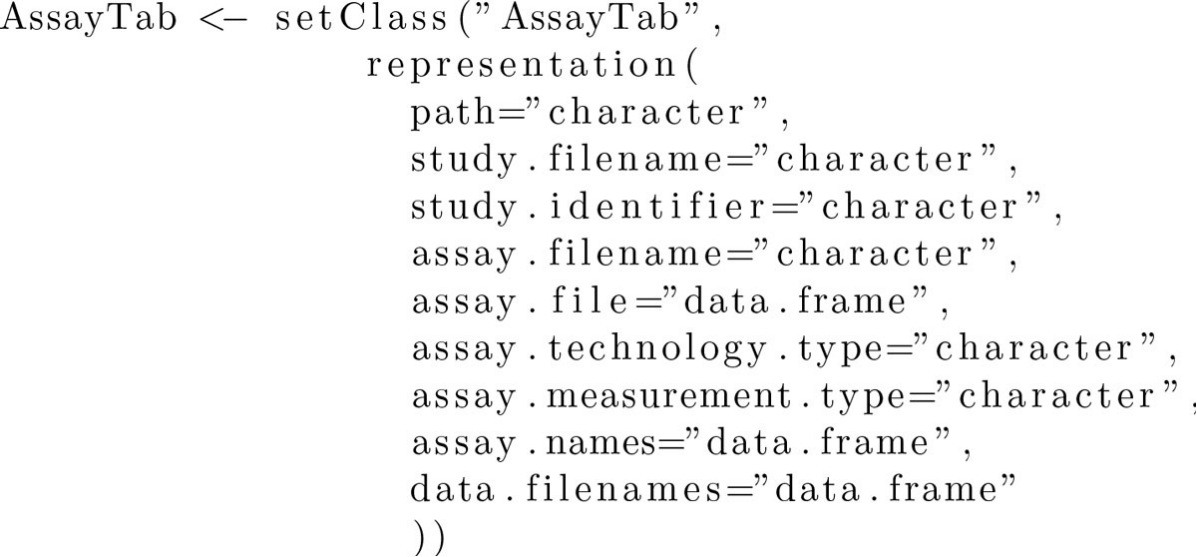
**Code for the AssayTab-class**.

The **Risa **package relies on the object-oriented programming capabilities of **R**, by defining S4 classes for the whole dataset ISAtab-class and for different types of assays, abstracted into a super-class AssayTab-class (see Figures 2 and 3 for the complete definition of these classes). The ISATab-class encapsulates the details of the experimental metadata expressed in the ISA-Tab format. Consequently, the class defines *fields*, or *slots *using the S4 terminology, for each of ISA syntactic elements. These slots contain, for example, the file system path where the ISA-Tab dataset can be found, the list of the declared *factors*, the *assay filenames *associated with the study, a vector with all the samples and so on.

**Risa**, exploiting the Biobase environment, defines the getAnnotatedDataFrameAssay method, which builds an AnnotatedDataFrame for a specific assay.filename available in an object of class ISATab-class. The resulting data structure corresponds to an entity formerly known as phenoData object, which holds all the phenotypic data and metadata, connecting sample names to all the factors and experimental variables associated with an assay on a row-based, record-like structure.

#### Building upon the ISA syntax: exposing core concepts of experimental design

The ISA-Tab syntax explicitly states multiple concepts related to the experimental design such as descriptors for the design type, the experimental factors --including their name and their values (referred to as *Study Factor Name *and *Study Factor Types*, respectively)--, and the study protocols.

Concepts as the experimental factors have a counterpart representation in **R **syntax, which are categorical variables that can take a limited number of values (the **R **'factor' data type). In this case, the **Risa **parsing stage builds factor variables for the declared factors in an ISA-Tab dataset, and these are part of the ISATab-class representation.

For example, parsing the faahKO data set metadata results in the creation of a factors slot corresponding to the ISA-Tab element *Factor Value[Genotype]*, which is the categorical variable whose levels are KO and WT, representing the knockout (treated) and wild-type (untreated) mice respectively. The factors slot is then populated with 6 KO and 6 WT elements, matching the replication level of the study.

In addition to those experimental design concepts available from the ISA syntax, the **Risa **package has been engineered to exploit the metadata to define new constructs not directly available. Considering factorial design, one of the extensions is the definition of *experimental treatments*, sometimes called *runs *in the statistical literature [30], which are formed by the combination of factor levels. We note that this definition refers to the statistical notion of *treatment*, which may not always coincide with the medical or biological notions of treatment.

In the faahKO dataset where only one factor is declared, the *treatments *slot is a list with one element, again related to the single assay, containing a factor containing only the two values KO and WT. As expected, this indicates that the two *treatments *applied to the mice are related to the distinction in genotype.

Another extension to the ISA syntax is the definition of *groups*, sometimes called *treatment groups *or *study groups*. The groups simply identify the set of samples corresponding to each of the *experimental treatments *for a particular assay. In the faahKO data set, there are two groups: one for the WT treatment and another for the KO treatment. The samples in each of these groups are: {*WT*_1_, *WT*_2_, *WT*_3_, *WT*_4_, *WT*_5_, *WT*_6_} and {*KO*_1_, *KO*_2_, *KO*_3_, *KO*_4_, *KO*_5_, *KO*_6_}.

For another example of the use of these definition for ISA-Tab datasets, the ARMSTRONG-S-3 study includes two factors (*Factor Value[hematopoietic progenitor cell type] *and *Factor Value[genetic modification]*) with three levels each (*"common myeloid progenitor"*,*"granulocyte macrophage progenitor"*,*"hematopoietic stem cell" *and *"Hoxa9/Meis1a fusion protein transduced HSC"*, *"murine stem cell virus (MSCV)-MLL-AF9 fusion protein transduced GMP"*,*"wild type"*). There are five experimental treatments, which consist of some of the combinations of the factor levels and five groups, each containing the samples for each of the treatments.

By providing the means to incorporate key information about the experimental design in the **R **objects resulting from the parsing of ISA archives, we aim to support a more intuitive way for users to manipulate their data.

##### Updating ISA metadata: incremental creation of metadata throughout the analysis pipeline

A key feature of managing experimental data is the ability to track the origin, or provenance, of the data files. The **Risa **package offers the function named updateAssayMetadata, taking a list of parameters as follows: an object of the type ISAtab-class, the filename of an assay file, a column name referencing the column to be modified and the list of values for that column. This method not only allows for updates of existing values in the assay file but also warrants addition of metadata. With a built-in function for metadata updates, the **Risa **package delivers capabilities supporting analysis provenance tracking under the ISA-Tab framework and its dedicated syntactic elements.

### Specialisations per assay type

The ISA format defines configurations [31] for a number of technologies (see Table 2) and the ISA infrastructure outlines several layout files for each of these technologies, supporting different use of the technique to specialised fields. For example, the mass spectrometry technology can be used for metabolomics or proteomics applications.

**Table 2:** Mapping ISA configurations to BiocViews

ISA Configuration File	ISA measurement	BiocViews	ISA technology	BiocViews
cellcount_flowcytometry	cell counting		flow cytometry	FlowCytometry

cellsorting_flowcyt	cell sorting		flow cytometry	FlowCytometry

clinical_chemistry	clinical chemistry analysis			

copynumvariation_micro	copy number variation profiling	CopyNumberVariants	DNA microarray	Microarray, aCGH

dnamethylation_micro	DNA methylation profiling	DNAMethylation	DNA microarray	Microarray, ChIPchip, CpGIsland, Methylseq

dnamethylation_seq	DNA methylation profiling	DNAMethylation	nucleotide sequencing	Sequencing, ChIPseq, CpGIsland, Methylseq

envgen survey_seq	environmental gene survey		nucleotide sequencing	Sequencing

genome_seq	genome sequencing		nucleotide sequencing	Sequencing

hematology	hematology			

heterozygosity_micro	loss of heterozygosity profiling	SNP, CopyNumber Variants	DNA microarray	Microarray

histology	histology			

histonemodification_seq	histone modification profiling	Regulation	nucleotide sequencing	Sequencing, ChIPseq

metaboliteprofiling_ms	metabolite profiling	Metabolomics	mass spectrometry	MassSpectrometry

metaboliteprofiling_nmr	metabolite profiling	Metabolomics	NMR spectroscopy	

metagenome_seq	metagenome sequencing		nucleotide sequencing	Sequencing

ppi_detection_micro	protein-protein interaction detection		protein microarray	Microarray

protein_dna_binding_ident_micro	protein-DNA binding site identification	Regulation	DNA microarray	Microarray, ChIPchip

protein_dna_binding_ident_seq	protein-DNA binding site identification	Regulation	nucleotide sequencing	Sequencing, ChIPseq

protein expression_ge	protein expression profiling	Proteomics	gel electrophoresis	

protein expression_micro	protein expression profiling	Proteomics	protein microarray	Microarray

protein expression_ms	protein expression profiling	Proteomics	mass spectrometry	MassSpectrometry, Proteomics

proteinident_ms	protein identification		mass spectrometry	MassSpectrometry, Proteomics

snpanalysis_micro	SNP analysis	SNP	DNA microarray	Microarray, GeneticVariability

studySample				

tfbsident_micro	transcription factor binding site identification	Regulation	DNA microarray	Microarray, ChIPchip

tfbsident_seq	transcription factor binding site identification	Regulation	nucleotide sequencing	Sequencing, ChIPseq

transcription_micro	transcription profiling	Transcription, GeneExpression	DNA microarray	Microarray, DifferentialExpression, ExonArray

transcription_rtpcr	transcription profiling	Transcription, GeneExpression, DifferentialExpression	real time PCR	qPCR

transcription_seq	transcription profiling	Transcription, GeneExpression	nucleotide sequencing	Sequencing, DifferentialExpression, RNAseq

While each of the technologies are associated with specific layout files, all of them involve material transformations (converting material into material or data files) and data transformations (converting raw data files into processed or derived data files). In order to deal with the multiplicity of assay types, the **Risa **package includes the class AssayTab-class, which is specialised for each technology type: MSAssayTab-class, MicroarrayAssayTab-class, SeqAssayTab-class, NMRAssayTab-class for mass spectrometry, microarray, sequencing and NMR spectroscopy assays, respectively.

Then, we exploit subtype polymorphism, or simply polymorphism, in object-oriented programming, to define generic functions such as getAssayRawDataFilenames and getAssayDerivedDataFilenames to retrieve the list of raw and processed data filenames per assay, respectively. For example, for MS assays, raw data files are *Raw Spectral Data Files *but for NMR spectroscopy assays, these are *Free Induction Decay Data Files*. Then, we define a generic function getRawDataFilenames applicable to the ISATab-class object, which relies on the assay-generic function to retrieve the raw data files for each type.

In the following sections, we detail how experiment metadata, once it has been extracted from ISA-Tab tables, can be accessed from other packages in a very straightforward manner.

#### Mass spectrometry assays: bridging from Risa to xcms

As stated above, one of the technologies supported by ISA is mass spectrometry (MS). From the **R**/Bioconductor side, the package *xcms *[32] includes several algorithms for processing mass spectrometry data. The package defines the xcmsSet-class, whose objects are built from the raw data files in the assay. The permissible formats for the raw data files are netCDF, mzData, mzXML and mzML [33]. Several other packages in support of mass-spectrometry analysis exist, for processing, visualisation, and statistical analysis: among those, *clippda*, *MassArray*, *MassSpecWavelet*.

Thus, with the purpose to provide a bridge between the ISA-Tab metadata and the analysis of mass spectrometry data, dedicated functions to deal with MS assays have been made available. The most important functionality is that which builds the xcmsSet object from the metadata, an object with two variants in the latest version of the package: processAssayXcmsSet.1factor and processAssayXcmsSet, considering the first factor and all factors in the assay, respectively.

In order to demonstrate the integration between the 2 packages, we ran, as an example, through a publicly available metabolomic datasets available from the Metaboligths [34] repository for metabolomics data at the European Bioinformatics Institute. The MTBLS2 dataset [35] was accessed using the **Risa **package to extract experimental metadata, build an xcmsSet, perform analysis using the xcms [32] and CAMERA [36] packages and, finally, augmenting the ISA archive with new metadata, i.e. the *Metabolite Assigment File *information resulting from the analysis process. Demonstration of this example is available in the supplementary material of the Metabolights article [10].

#### Microarray assays: bridging from Risa to affy

DNA microarray is another technology supported in the ISA infrastructure, where configuration files ensure compliance with the Minimum Information for Microarray Experiments (MIAME) standard [37]. The Biobase package includes the MIAME class to encapsulate the metadata, and in **Risa**, the method getMIAMEMetadata allows to build MIAME objects directly from the ISA-Tab metadata.

A popular platform for DNA microarray is *Affymetrix *and the affy package [38], available since BioC version 1.6 (R-2.1), provides classes and methods for storing, managing and analysing oligonucleotide arrays in the *Affymetrix *platform. The package relies on elements from the Biobase package, such as the ExpressionSet class, which encapsulates high-throughput assays with their experimental data.

We rely on the justRMA method in affy, to read CEL files associated with the assay and compute an expression measure as an ExpressionSet using the Robust Multi-array Average (RMA) normalization method. The **Risa **method is called getExpressionSet and receives an ISATab-class object and the microarray assay filename as parameters.

### Recommending Bioconductor packages: contextual awareness

Bioconductor requires that each submitted package be annotated or tagged with one or more BiocViews [39] categories in order to aid in the classification and retrieval of Bioconductor packages. BiocViews is itself an **R**/Bioconductor package whose sole purpose is to categorise **R **package repositories and produce dedicated HTML pages. The categories are defined without any ontological framework and, while reasonably explicit, their interpretation is left to the users and submitters. So, the classification of packages is, *in-fine*, dependent on the interpretation of each package owner.

**Figure 4:**
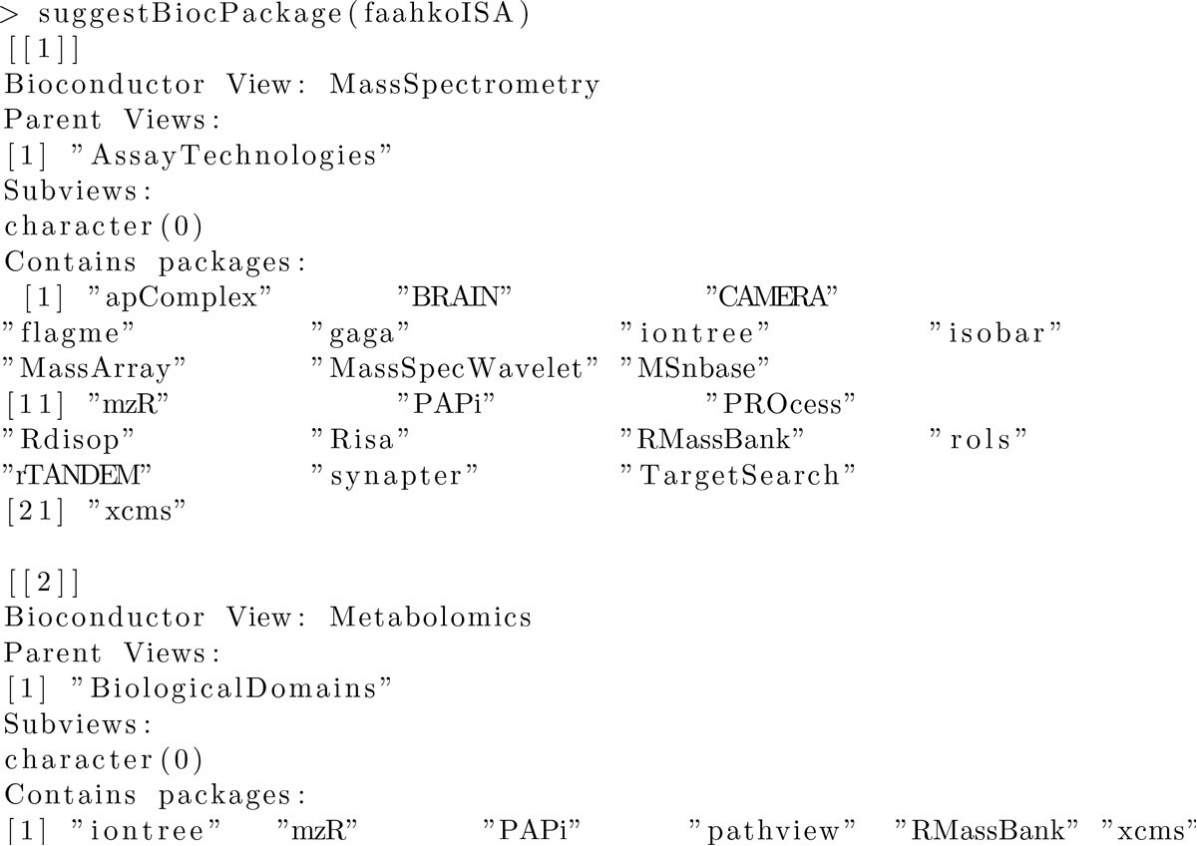
**Output of suggestBiocPackage(faahkoISA)**.

In **Risa**, we suggest potentially relevant Bioconductor packages for a given ISA-Tab dataset relying on the BiocViews classification. This functionality can be used to guide researchers in identifying suitable BioC packages but can also be used to support training and education by familiarising newcomers to popular analysis packages. To reach this goal, a mapping between the ISA technology and measurement types, as given in the ISA Configuration-Files [31], and the current BiocViews categories [39] was performed. The results are included in Table 2. Given an object from the ISAtab-class, the technology and measurement types serve as the basis for the function suggestBiocPackage to retrieve a list of appropriate packages related to the assays in the data set. To further refine the filtering and selection process, a specific BioC version to be considered can be included as input parameter. As an example, the output for the faahKO dataset is presented in Figure 4.

### Usage

**Figure 5:**
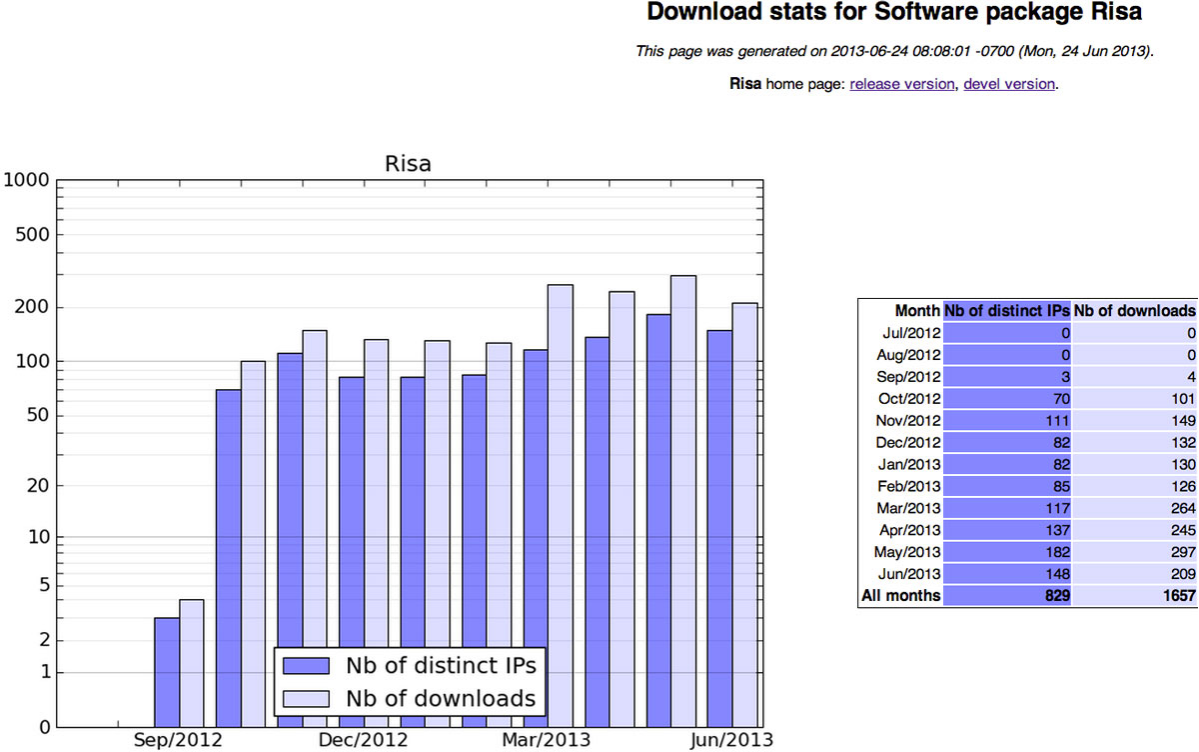
**Risa usage**. Download statistics for the Risa package in Bioconductor retrieved on 24*^th ^*June 2013, latest data available at http://bioconductor.org/packages/stats/bioc/Risa.html.

Since its first release in Bioconductor version 2.11, which was announced in September 2012, the **Risa **package has been downloaded from http://bioconductor.org, and without considering other mirroring sites, a total number 1657 times and accessed from 829 different IP addresses up to 24*^th ^*June 2013 (see Figure 5). These statistics, available through the Bioconductor website, are testament of the popularity of the ISA-Tab metadata tracking framework, which addresses the need of offering a common syntax encompassing study design related elements while specialising when needed at assay level, alleviating the problem of siloed metadata formats.

## Discussion

We have presented **Risa**, an **R**/Bioconductor module to facilitate the integrative analysis of multi-omics experiments whose metadata is ISA-Tab formatted. The ISA-Tab syntax details a hierarchical structure ranging from general information about the investigation -- general objectives, personnel involved, publications, experimental factors, protocols -- to granular information about study experimental units, to details on the analytical measurements and data files, organised in sets of tables hinged around biological materials used as input to data collection processes.

We described the functionalities of the **Risa **package and its application to several use cases anchored to different technologies: mass spectrometry and DNA microarray. Each of the use cases consisted in building **R **objects using **Risa **as the bridge between the metadata and further processing with relevant **R**/Bioconductor packages for each of the technologies. The goal of these use cases is to highlight how an integrative analysis can be driven by the information on the metadata files.

The package's main function is to act as servant to metadata extraction and persistence in the context of an analysis framework. To close the metadata tracking loop, **Risa **offers functions to capture data analysis results back to ISA-Tab format by logging newly generated data files and methods. Collaboration with Metabolights public repository validates the approach by including **Risa **parsing into the chain of data custody established by the resource.

Finally, to support scientists in their exploration of results, **Risa **features a context-aware package recommendation function. The function relies on ISA assay metadata and BiocViews package annotations. Future directions of work include streamlining workflow integration for flow cytometry data, NMR data and RNA-Seq data --using, for instance, the flowCore Bioconductor package [40], the BATMAN package [41] and the DESeq package [42], respectively--, and allowing users to save their **R **analysis script as a ISA protocol back to the archive. We aim to explore the automation of this process. Finally, following ISA reliance on ontology terms supplied by the Ontology of Biomedical Investigations (OBI) [43], providing a similar semantic backing to biocViews categories could prove worth exploring with the **R **community.

## Competing interests

The authors declare that they have no competing interests.

## Authors' contributions

AGB developed the **Risa **package, followed the procedures for submission to Bioconductor and is responsible for maintaining the package. PRS provided guidance and example ISA-Tab datasets throughout the development process. SN provided suggestions for the code functionality and contributed to methods dealing with mass spectrometry data and interface to the *xcms *package. AGB wrote the manuscript based on PRS's structure and input and all the authors provided edits and feedback. EM produced Figure 1. PRS, EM and SAS provided input on ISA related background and information. SAS and PRS provided vision and scope. All authors have revised, read and approved the final manuscript.

## Declarations

AGB, EM, SAS and PRS would like to thank their funding support to BBSRC BB/I000771/1, BB/I025840/1 and BB/J020265/1, EU COSMOS EC312941 and the University of Oxford e-Research Centre.

This article has been published as part of *BMC Bioinformatics *Volume 15 Supplement 1, 2014: Integrated Bio-Search: Selected Works from the 12th International Workshop on Network Tools and Applications in Biology (NETTAB 2012). The full contents of the supplement are available online at http://www.biomedcentral.com/bmcbioinformatics/supplements/15/S1.
